# Generation time-out grows up: young adults’ reports about childhood time-out use and their mental health, attachment, and emotion regulation

**DOI:** 10.1007/s00787-024-02408-8

**Published:** 2024-03-05

**Authors:** Jingyi Xu, Lucy A. Tully, Mark R. Dadds

**Affiliations:** The University of Sydney, Camperdown, Sydney, AU, NSW 2008 Australia

**Keywords:** Child mental health, Parent discipline, Time-out, Trauma

## Abstract

Time-out (TO) is a widely utilised parental discipline technique with a strong evidence-base that nonetheless has attracted controversy regarding potential adverse effects on mental health in developing children. Associations between TO implementation and mental health outcomes have rarely been investigated, especially through the eyes of children who grew up experiencing TO. This study recruited 407 university students (Study 1) and a community sample of 535 young adults (Study 2); both samples aged 18–30 years. Young adults were surveyed on their retrospective reports of childhood TO experience, childhood experiences of adversity, perceived parenting style and parental attachment, and their current mental health outcomes (attachment style, emotion regulation and mental health). In Study 1, 334 (82.1%) young adults reported experiencing TO in childhood, but with widely varied implementation that differed considerably from its evidence-based ideal. Reports of more TO appropriate implementation were associated with less avoidant attachment, better mental health, and emotion regulation, over and above the effects associated with authoritative parenting and secure attachment in childhood. While exposure to childhood adversity was associated with poorer adulthood outcomes, TO implementation did not moderate the association. Study 2 replicated most findings from Study 1, except that appropriate TO implementation displayed a positive association with mental health and no associations with anxious and avoidant attachment and emotion regulation. These findings suggest the safety of TO use with young children, including those who experienced childhood adversity, and highlight the importance of disseminating sufficient parenting information on TO in the community.

**Supplementary Information:**

The online version contains supplementary material available at 10.1007/s00787-024-02408-8.

## Introduction

Time-out (TO) is a widely used and evidence-based parental discipline technique that has attracted debate concerning its potential adverse effects on child mental health. TO, short for time-out from positive reinforcement, is defined as a temporary removal from a reinforcing environment or stimuli (e.g., parental attention) for a short separation period contingent on behaviours targeted for reduction [1]. It was introduced in the 1960s as an alternative to physical and emotionally abusive forms of punishment [2], and it is now one of the most broadly utilised discipline techniques across countries and demographic characteristics [3–5]. Typical behaviours targeted are verbal and physical aggression, noncompliance, rule-breaking, and temper tantrums. Its theoretical foundation stems primarily from social learning theory, where proponents suggest TO as both a punisher and an extinction procedure that extinguishes negative behaviours through removing previously reinforced stimuli (e.g., parental attention) contingent on misbehaviour [1]. Use of TO is premised on ‘time-in’ being characterised as a wealth of positive reinforcement and engagement within a warm and loving parenting context. Despite the seeming simplicity in TO procedures, it is a complicated and stepped strategy that requires high-level implementation fidelity according to its evidence-based parameters (e.g., location, consistency, immediacy of implementation). While there is evidence suggesting widely varied and inconsistent implementation of TO in the community [4, 6], the effectiveness and safety of TO are thought to be highly dependent on the degree of adherence to its evidence-based parameters, also referred to as the appropriateness of TO implementation [1, 7].

Over the past few decades, opposing voices have questioned the safety of TO use on young children. Critics often claim TO as developmentally inappropriate and overly punitive, leading to impaired parent-child attachment and diminished mental health and emotion regulation abilities in children [8–10]. Such controversies are especially contentious around children with adverse histories as some opponents suggest that TO triggers retraumatisation in evoking emotional pain in children and serves as a reminder of past traumatic events, or that TO itself is traumatising in promoting a sense of rejection and distress that subsequently produces pain and suffering equivalent to physical pain [8, 10]. Interestingly, while opponents consider TO as a negative discipline that is based on imposition of parental control and isolation of the child [6, 11], TO is conceptualised by its proponents as a positive parenting strategy that provides children with a safe and reassuring context to acquire calmness and regulate [1, 12]. As such, proponents suggest use of TO could help children regain emotional control and learn self-discipline, and extinguish the emotional distress associated with previous adverse events from an exposure perspective [1, 13].

Given the pervasive use of TO and the ongoing concerns, it is important for more and better-quality research to investigate its long-term impacts. There has been overwhelming evidence from experimental studies and reviews demonstrating the short-term effectiveness and safety of TO use alone [7, 14, 15] and as a component in effective parenting programs based on findings from randomised controlled trials and meta-analyses [16–19], including parenting interventions for traumatised children [20, 21]. In a recent longitudinal study examining the relationship between TO use and child attachment and emotional outcomes, TO displayed no significant associations with parent-child relationships and long-term negative emotional and behavioural outcomes in children [22]. In contrast, only one study has demonstrated a harmful effect of TO in exhibiting a small significant correlation (*r* = 0.13) with greater anxiety symptoms in children [23]. Such a finding is consistent with the idea that TO might interfere with attachment bonds and emotion regulation; however, it is difficult to interpret as the study did not define TO.

To date, research on TO has neglected the critical aspect regarding the appropriateness of TO implementation. Further, no studies have examined the long-term effects of TO use beyond childhood, especially through the eyes of children who grew up experiencing TO; and no research has examined young adults’ perceptions of TO and its effects on their current mental health. Importantly, given the lack of empirical evidence showing TO is associated with poor outcomes when used with children with trauma histories [1], no study has directly examined TO use in relation to the level of adversity individuals experienced in childhood. Here we report on the first examination of retrospective reports of appropriate childhood TO use in two samples of young adults.

Research hypotheses were as follows: (1) TO was predicted to be widely used in childhood according to young adults’ self-reports; however, wide variations in the adherence to evidence-based implementation parameters were expected; (2) more appropriate implementation of TO was predicted to associate with better mental health outcomes in young adults, over and above the effect afforded by the parenting context; (3) appropriate TO implementation was hypothesised to act as a moderator that alleviated the negative effect of childhood adversity on young adults’ mental health outcomes.

## Study 1

### Methods

#### Participants and procedure

This study sampled 419 undergraduate psychology students aged 18 to 30 years. Participants were recruited via the research participation system at the University of Sydney and participated in exchange for course credit. The survey was conducted via an online survey platform, Qualtrics, from June to August 2021. Participants voluntarily signed up to the study and provided written consent before participation. The questionnaire order was counterbalanced with either baseline or retrospective measures presented first, and participants were randomly assigned to one of the counterbalanced surveys. Of the 419 participants who completed the study, 12 were excluded due to providing random or duplicated responses. Of the remaining 407 participants, 306 (75.2%) were females and the mean (SD) age was 19.86 (2.03) years. None of the participants were parents. This study followed the Strengthening the Reporting of Observational Studies in Epidemiology (STROBE) reporting guideline.

### Measures

#### Implementation of time-out scale (ITO)

Retrospective reports of appropriate TO use were assessed using the modified ITO [7]. As the original ITO is a parent report measure, modifications involved wording changes to a retrospective measure for young adults. This scale consists of 13 items to measure the usage and appropriateness of TO implementation during the age of 2 to 8 years. Specifically, one item measured the frequency of TO use on a 5-point scale ranging from *Never* to *Always (i.e., “How often did your parent(s)/caregiver(s) use time-out when you misbehaved as a child”)*, and twelve items formed the implementation scale based on eleven evidence-based parameters (see Online Resource 1). Higher scores indicate more appropriate TO implementation. The ITO exhibits good convergent validity through concurrent parent reports of TO use [7].

### Subjective perceptions of TO

Given there is no existing measure, a 2-part measure was developed to examine young adults’ perceptions of TO. The first part involved seven items: one item assessed participants’ perceived effectiveness of TO as a general parenting strategy on a 5-point scale ranging from *Very Ineffective* to *Very Effective* and six items examined the extent to which participants perceived childhood use of TO, and other discipline strategies apart from TO, as contributing to their current mental health outcomes on a 5-point scale ranging from *Major Negative Impact* to *Major Positive Impact*. The second part involved ten items where participants were asked to imagine themselves parenting in two given scenarios and rate the acceptability of five discipline techniques (smacking, yelling, TO, taking away a privilege, and shaming) on a 5-point scale ranging from *Not at all Appropriate* to *Very Appropriate*.

#### Adulthood mental health outcomes

Mental health outcomes of attachment style, emotion regulation, and mental health were measured using the Adult Attachment Scale (AAS) [24], the Difficulties in Emotion Regulation Scale (DERS) [25], and the K10 [26]. Details of these measures are provided in Online Resource 2.

#### Parenting context and other measures

Perceived parenting styles and parental attachment were measured using the Parenting Styles and Dimensions Questionnaire–Intergenerational Version (PSDQ-G1) [27] and Descriptions of Parental Caregiving Style (DPCS) [28]. Exposure to childhood adversity was assessed through the Adverse Life Experience Scale (ALES) [29]. Sociodemographic characteristics including age, gender, parental and relationship status, and composition of childhood caregivers were examined. Based on the family composition, participants rated the quality of their current relationship with their female/male caregiver on a 9-point Likert scale ranging from *Very Poor* to *Excellent*. Participants also rated the accuracy of recollection on a 5-point scale ranging from *Not at all Accurate* to *Very Accurate*. Detailed descriptions of the measures are available in Online Resource 2.

### Statistical analysis

An *a priori* power analysis was performed for linear regression using G Power 3.1.9.6 [30]. Based on a moderate effect size found from a previous study investigating the association of parent-reported TO use with children’s attachment and mental health [7], this study expected a small to moderate effect size due to its retrospective nature. Using a power of 0.80 [7], with α = 0.05, a minimum sample size of 143 was required for detecting a small to moderate effect size of F^2^ = 0.01. All analyses were conducted using IBM SPSS Statistics Version 26. To maintain an experimental-wise significance threshold of 0.05, with 3 dependent variables, all hypotheses were tested at *p* < 0.01. All tests were 2-tailed. Chi-square tests of independence, univariate analysis of variance (ANOVA) and regressions were used to determine if any sociodemographic variables or perceptions of quality of the current relationship with caregivers needed to be controlled in the main analyses. Descriptive statistics were computed to examine the frequency and appropriateness of TO implementation, perceived TO effectiveness and impacts. A one-way repeated measure ANOVA was conducted to investigate the acceptability of TO compared to other discipline strategies. Bivariate correlations were examined to assess the associations between childhood TO use, young adults’ perceptions of TO, childhood adversity and parenting context. Participants with no experience of TO were excluded from the remaining analyses.

Three multivariate hierarchical regressions were conducted to examine the mean-adjusted associations between appropriate TO implementation and each dependent variable (mental health, emotion regulation and attachment). In all regression models, with adulthood adversity, childhood secure attachment, authoritative and authoritarian parenting entered as covariates, appropriateness of TO implementation and childhood adversity were entered in the first step. To determine the moderating effects of TO, an interaction term between appropriate TO implementation and childhood adversity was computed and entered as the second step. Since accounting for both paternal and maternal parenting contexts (comprising authoritative and authoritarian parenting styles and parental attachment in childhood) would limit the sample to individuals with both parents as primary caregivers, the analyses above were performed with maternal parenting context as a covariate and then repeated with both maternal and paternal parenting contexts as covariates, and it would be noted if hypotheses testing differed.

## Results

Overall, 229 participants (56.3%) reported being exposed to at least one childhood adversity. Participants reported the following compositions of childhood caregivers: both parents (56.5%), mother (34.9%), father (2.2%), and others (6.4%). Three hundred and twenty-three participants (79.4%) indicated their recollection of childhood experiences as moderately to very accurate. As findings did not differ according to the inclusion of participants who rated their recollections more accurately, results are reported for the entire sample. No significant differences in TO frequency and young adults’ mental health outcomes were found as a function of age, gender, and composition of or current relationship with childhood caregivers.

### Frequency and appropriateness of TO implementation

As predicted, TO was widely used as a discipline strategy: 334 young adults (82.1%) reported experiencing TO in childhood, and 85 (20.9%) reported TO being frequently or always used by their caregiver(s). As anticipated, although TO was commonly used, adherence to evidence-based parameters in implementation was widely varied (Table 1). For example, only 76 (22.8%) participants reported that their parents were calm when delivering TO, and 104 (31.1%) indicated that TO was delivered immediately contingent on misbehaviours.

**Table 1:** Percentages of adherence to the evidence-based parameters of time-out^a^

Evidence-based parameter	n	%
TO use on operant child behaviours^b^	238	71.3
Consistent implementation	180	53.9
Immediate TO use contingent on misbehaviour	104	31.1
Calm delivery of TO	76	22.8
Appropriate TO location with minimal reinforcement	149	44.6
Minimal parental attention during TO	170	50.9
Enforcing a back-up consequence to TO escape	195	58.4
Parent monitored TO release	215	64.4
Contingent release on calm behaviour	152	45.5
Parent-child reconciliation after TO	169	50.6
Restatement of the original command	197	59.0

^a^ TO = time-out

^b^ Values were calculated as the average of the items on the parameter

### Perceptions of TO

While 176 (43.2%) young adults endorsed TO as effective, 129 (31.7%) perceived TO as neither effective nor ineffective and 102 (25.1%) regarded TO as ineffective. TO was on average rated as a neutral discipline technique compared to other discipline techniques such as taking away a privilege or yelling (Table 2). In addition, although over half of the young adults regarded TO as having no impact on their current attachment and mental health, 136 (40.7%) reported positive impacts of TO on their current emotion regulation ability. In contrast, while childhood use of other discipline strategies was perceived to have no impact on young adults’ current attachment (36.1%), it was endorsed as having predominately negative impacts on mental health (46.9%), and emotion regulation (42.3%) (Online Resource 3). Finally, while no significant association was found between appropriateness of TO and its perceived impacts, more appropriate TO implementation was positively correlated with greater perceived effectiveness (*r* = 0.16 [95% CI 0.06–0.26], *p* < 0.01) and acceptability (*r* = 0.15 [95% CI 0.04–0.26], *p* < 0.01).

**Table 2:** Descriptive statistics for discipline acceptability ratings^a^

Discipline technique	Mean	SD	95% CI
Take away a privilege	3.25^b^	1.28	3.13–3.38
Time-out	2.93^b^	1.25	2.81–3.05
Yelling	1.94^b^	0.99	1.85–2.04
Smacking	1.55^b^	0.67	1.47–1.64
Shaming	1.38^b^	0.85	1.30–1.43

^a^ Acceptability scores were rated on a 5-point scale ranging from *Not at all acceptable* to *Very acceptable*

^b^ Means with the same superscript are significantly different from each other at *p* < 0.01

### Associations of appropriateness of TO implementation, childhood adversity and mental health outcomes

As results appeared unchanged when controlled for maternal parenting contexts or both maternal and paternal parenting contexts, results based on maternal parenting contexts are reported due to the larger sample size.

Childhood adversity was significantly correlated with worse mental health outcomes (avoidant attachment, *r* = 0.23 [95% CI 0.11–0.35], *p* < 0.01; anxious attachment, *r* = 0.15 [95% CI 0.03–0.27], *p* < 0.05; emotion dysregulation, *r* = 0.34 [95% CI 0.19–0.46], *p* < 0.01; mental health problems, *r* = 0.25 [95% CI 0.08–0.40], *p* < 0.01). However, in the regression analysis, irrespective of exposure to adulthood adversity, appropriateness of TO use and the parenting context, childhood adversity was only significantly associated with adulthood emotion dysregulation, but not attachment and mental health problems. As hypothesised, the main effects of appropriate TO implementation were significant, exhibiting positive associations with better mental health outcomes in young adults over and above the effects afforded by the parenting context (Table 3). Specifically, regardless of the exposure to childhood adversity, adulthood adversity and parenting context, for each point increase in appropriate TO implementation score, scores on avoidant attachment, emotion dysregulation, and mental health problems were predicted to decrease by 0.32 points, 0.81 points, and 0.24 points, respectively. However, despite the negative association as predicted, appropriate TO implementation was not significantly associated with more anxious attachment. Contrary to predictions, appropriate TO implementation did not moderate the negative associations between childhood adversity and young adults’ mental health outcomes.

**Table 3:** Adjusted associations between appropriateness of time-out implementation and childhood adversity with insecure attachment, emotion dysregulation and mental health problems in study 1^a^

Dependent variable	Independent variable	B	SE	95% CI	*p*	*sr* ^*2 b*^
Avoidant attachment	*Step 1 (R*^*2*^ *= 0.212)*					
	Childhood adversity	0.15	0.20	-0.24 to 0.54	0.457	0.00
	Appropriate TO implementation	-0.32	0.11	-0.54 to -0.10	0.004	0.04
	*Step 2 (R*^*2*^ *= 0.212)*					
	Childhood adversity	0.15	0.20	-0.25 to 0.54	0.462	0.00
	Appropriate TO implementation	-0.32	0.11	-0.54 to -0.10	0.004	0.04
	Childhood adversity by Appropriate TO implementation	-0.002	0.03	-0.06 to 0.06	0.945	0.00
Anxious attachment	*Step 1 (R*^*2*^ *= 0.133)*					
	Childhood adversity	0.11	0.13	-0.15 to 0.36	0.413	0.00
	Appropriate TO implementation	-0.15	0.72	-0.29 to -0.01	0.034	0.03
	*Step 2 (R*^*2*^ *= 0.136)*					
	Childhood adversity	0.11	0.13	-0.14 to 0.37	0.380	0.00
	Appropriate TO implementation	-0.15	0.07	-0.29 to -0.01	0.035	0.03
	Childhood adversity by Appropriate TO implementation	-0.015	0.18	-0.02 to 0.06	0.400	0.00
Emotion dysregulation	*Step 1 (R*^*2*^ *= 0.250)*					
	Childhood adversity	1.25	0.43	0.41 to 2.09	0.004	0.02
	Appropriate TO implementation	-0.81	0.22	-1.24 to -0.37	<0.001	0.03
	*Step 2 (R*^*2*^ *= 0.250)*					
	Childhood adversity	1.25	0.43	0.41 to 2.10	0.004	0.02
	Appropriate TO implementation	-0.81	0.22	-1.25 to -0.37	<0.001	0.03
	Childhood adversity by Appropriate TO implementation	0.002	0.06	-0.12 to 0.12	0.979	0.00
Mental health problems	*Step 1 (R*^*2*^ *= 0.212)*					
	Childhood adversity	0.26	0.14	-0.02 to 0.53	0.064	0.01
	Appropriate TO implementation	-0.24	0.07	-0.38 to -0.10	0.001	0.03
	*Step 2 (R*^*2*^ *= 0.216)*					
	Childhood adversity	0.27	0.14	-0.01 to 0.54	0.059	0.01
	Appropriate TO implementation	-0.23	0.07	-0.38 to -0.09	0.002	0.03
	Childhood adversity by Appropriate TO implementation	0.02	0.02	-0.02 to 0.06	0.241	0.00

^a^ TO = time-out. Regression models controlled for adulthood adversity and maternal parenting context (not shown)

^b^ Squared semi-partial correlation, representing the unique variance accounted for by each variable

## Study 2

Study 2 tested the generalisability of the previous findings in a community sample of young adults. Hypotheses were the same as Study 1.

### Methods

In total, 535 participants aged 18–30 years (*M*_age_ = 23.89, *SD* = 3.74) residing in Australia were recruited through an independent online research panel, Qualtrics. Of these, three hundred and fifty-eight (66.9%) participants were females and 190 (35.5%) were identified as parents. The only inclusion criterion was an education level of below tertiary level. The study was conducted between December 2021 and February 2022. All other aspects of the method are identical to Study 1.

## Results

The compositions of primary caregivers in childhood were reported as follows: both parents (44.9%), mother (49.5%), and father (5.6%). Overall, 364 participants (68.0%) were exposed to at least one childhood adversity and 416 (77.8%) rated their recollection as moderately to very accurate. Findings did not change according to the inclusion of participants with greater recollection accuracy. Results also did not differ depending on participants’ age, gender, parental status, and composition of or current relationship with childhood caregivers. Overall, 465 young adults (86.9%) reported experiencing TO as a child. As Study 2 largely replicated the previous findings, full results of Study 2 are presented in Online Resource 4.

While displaying consistent positive bivariate correlations with worse mental health outcomes (avoidant attachment, *r* = 0.26 [95% CI 0.17–0.35], *p* < 0.01; anxious attachment, *r* = 0.27 [95% CI 0.18–0.36], *p* < 0.01; emotion dysregulation, *r* = 0.36 [95% CI 0.29–0.45], *p* < 0.01; mental health problems, *r* = 0.34 [95% CI 0.26–0.43], *p* < 0.01), childhood adversity was not significantly associated with mental health outcomes in the regressions. As shown in Table 4, as expected, appropriateness of TO implementation was significantly associated with better mental health over and above the effects accounted by parenting context. That is, irrespective of childhood adversity, adulthood adversity and parenting context, for each point increase in appropriate TO implementation score, mental health problem score was predicted to decrease by 0.22 points. However, contrary to predictions, appropriateness of TO implementation was not associated with anxious and avoidant attachment and emotion dysregulation in adulthood. Further, opposing hypotheses, appropriate TO implementation did not moderate the negative associations of childhood adversity with mental health outcomes.

**Table 4:** Adjusted associations between aappropriateness of time-out implementation and childhood adversity with insecure attachment, emotion dysregulation and mental health problems in study 2^a^

Dependent variable	Independent variable	B	SE	95% CI	*p*	*sr* ^*2 b*^
Avoidant attachment	*Step 1 (R*^*2*^ *= 0.260)*					
	Childhood adversity	0.01	0.07	-0.12 to 0.13	0.914	0.00
	Appropriate TO implementation	- 0.01	0.07	-0.33 to 0.14	0.921	0.00
	*Step 2 (R*^*2*^ *= 0.260)*					
	Childhood adversity	0.002	0.07	-0.13 to 0.13	0.974	0.00
	Appropriate TO implementation	-0.01	0.07	-0.15 to 0.14	0.238	0.00
	Childhood adversity by Appropriate TO implementation	-0.005	0.01	-0.02 to 0.01	0.570	0.00
Anxious attachment	*Step 1 (R*^*2*^ *= 0.218)*					
	Childhood adversity	0.01	0.04	-0.08 to 0.10	0.788	0.00
	Appropriate TO implementation	- 0.12	0.05	-0.22 to 0.02	0.017	0.02
	*Step 2 (R*^*2*^ *= 0.218)*					
	Childhood adversity	0.01	0.04	-0.08 to 0.10	0.769	0.00
	Appropriate TO implementation	-0.12	0.05	-0.22 to 0.02	0.017	0.02
	Childhood adversity by Appropriate TO implementation	-0.001	0.01	-0.01 to 0.01	0.990	0.00
Emotion dysregulation	*Step 1 (R*^*2*^ *= 0.246)*					
	Childhood adversity	0.33	0.18	-0.02 to 0.68	0.064	0.01
	Appropriate TO implementation	-0.21	0.20	-0.60 to 0.20	0.314	0.00
	*Step 2 (R*^*2*^ *= 0.253)*					
	Childhood adversity	0.42	0.18	0.06 to 0.78	0.022	0.01
	Appropriate TO implementation	-0.19	0.20	-0.59 to 0.21	0.344	0.00
	Childhood adversity by Appropriate TO implementation	0.05	0.03	0.002 to 0.10	0.042	0.01
Mental health problems	*Step 1 (R*^*2*^ *= 0.238)*					
	Childhood adversity	0.10	0.07	-0.03 to 0.23	0.124	0.01
	Appropriate TO implementation	-0.22	0.07	-0.37 to -0.08	0.003	0.02
	*Step 2 (R*^*2*^ *= 0.239)*					
	Childhood adversity	0.11	0.07	-0.02 to 0.25	0.085	0.01
	Appropriate TO implementation	-0.22	0.07	-0.37 to -0.07	0.003	0.02
	Childhood adversity by Appropriate TO implementation	0.01	0.01	-0.01 to 0.03	0.340	0.00

^a^ TO = time-out. Regression models controlled for adulthood adversity and maternal parenting context (not shown)

^b^ Squared semi-partial correlation, representing the unique variance accounted for by each variable

## Discussion

This study was the first to examine the associations of reports of childhood TO experience with mental health in young adults. Overall, appropriate implementation of TO exhibited no negative associations with deteriorated mental health outcomes across the two studies. Instead, in Study 1, reports of appropriate TO implementation in childhood were positively associated with enhanced adulthood mental health, emotion regulation and attachment, above and beyond the effects afforded by the broader parenting context. Moreover, appropriateness of TO implementation did not moderate the negative associations of childhood adversity with young adults’ mental health outcomes in both studies.

TO is a prevalent discipline strategy with most young adults reporting having experienced parental use of TO in childhood at comparable rates to past studies [7, 31]. However, similar to findings in previous research [4, 6], parents were implementing TO in an inconsistent, emotional, and non-contingent manner that highly diverged from the evidence-based guidelines. The lack of widespread implementation fidelity is concerning, especially in light of the positive associations found between inappropriate TO implementation and diminished mental health outcomes in young adults. As suggested by proponents, emotional, unpredictable and unreasonable implementation of TO can likely damage the parent-child attachment bond and interfere with children’s ability to regulate their emotions, leading to coercive parent-child interactions and reduced effectiveness of TO [6]. Together, these findings provide evidence that concerns about the safety of TO are likely attributable to widespread misunderstanding and poor implementation in the community. It is thus critical for clinicians and health care providers to disseminate parenting resources on evidence-based TO procedures, thereby empowering parents to utilise TO with greater fidelity.

Regarding the perceptions of TO, young adults generally endorsed TO as an effective and acceptable discipline strategy that had more positive impacts on their current well-being than alternative disciplines used in childhood. Interestingly, for those who experienced TO, more appropriate use was positively correlated with greater perceived effectiveness and acceptability, further indicating that the appropriateness of TO implementation is a vital aspect that relates to how children view TO as a discipline strategy. These results can be contrasted with past findings suggesting that TO is perceived as aversive and punitive by young children without examining the appropriateness of implementation [9].

Overall, reports of appropriate use of TO displayed no to positive associations with mental health outcomes in the long term and aligned with the abundant research suggesting the safety and beneficial effects of TO on children’s attachment and emotional development [15, 22]. In addition, appropriateness of TO implementation did not moderate the harmful effects of childhood adversity on young adults’ current outcomes, thus not supporting concerns regarding the deleterious effects of TO on children with adverse histories [8, 10]. Contrary to the advocation of TO as traumatising to children and diminishing the well-being of those with histories of adversity, the present findings have provided evidence supporting appropriate implementation of TO as a safe and potentially beneficial technique that maintains secure attachment and enhances child mental health regardless of children’s adverse experiences.

### Strengths and limitations

The current findings should be interpreted in terms of the following strengths and limitations. One notable achievement of this study is the novel insight into the long-term impacts of TO revealed by utilising retrospective measures. However, the retrospective nature of this study also serves as the most dominant limitation, as retrospective reports of childhood experiences are often fallible, imperfect and limited by memory. Despite the attempt to control for the effects of recall accuracy to maximise the validity of the results, findings should be interpreted with caution as the accuracy and reliability of childhood recollections are likely hampered by various internal and external factors throughout young adults’ development [32]. Moreover, the reliance on self-report data indicates that the results might be influenced by social desirability bias. Findings were also likely subject to single informant bias, whereby the idiosyncrasies of young adults likely inflated the correlations between measures. Also, it should be noted that the study was not pre-registered. Finally, the cross-sectional nature of this study prohibits inferences of causality or directionality to be drawn.

Together, these limitations prompt future studies to employ a multi-informant design, such as seeking corroborative evidence from parents or siblings, to allow for more objective and accurate accounts of childhood experiences. Where feasible, future research should utilise longitudinal designs to assess the causal effects of appropriate TO use as a predictor of child development and mental health, thereby further ascertaining the safety of TO use for young children with varying levels of exposure to adversity. Additionally, future research should recruit adversely affected clinical samples that are more likely to exhibit significant behavioural and trauma-related issues to improve the generalisability of the findings to clinical populations.

### Implications

In conclusion, utilising young adults’ retrospective reports of childhood TO use, the present study was unable to find any evidence in support of the contentions regarding the deleterious impacts of TO on broader child mental health. Instead, findings aligned with the ample evidence suggesting TO as a safe and appropriate discipline strategy that is associated with enhanced mental health outcomes in the long term, even amongst those who suffered childhood adversity. There are several implications of the findings for parents and practitioners who work with parents to deliver evidence-based parenting strategies for child mental health problems. First, parents who choose not to use TO and clinicians who advocate against its use, due to its potential harmful effects, should reconsider the TO strategy given the overwhelming evidence supporting its effectiveness and the positive effects on child development. Particularly, mental health professionals who are disseminating public resources against TO and adopting it as a policy should reconsider their position, given their recommendations are clearly not evidence-based. Second, since this study found no evidence that the opponents’ concerns are warranted, opponents should re-examine and potentially reframe their claims as cautioning against the harmful effects associated with inappropriate or punitive use of TO. Third, researchers and clinicians should widely disseminate information to parents on how to implement TO according to evidence-based guidelines. This information is not readily available in the community or commonly used digital parenting apps [33, 34]. Internet-based information and campaigns are needed to better educate parents about the evidence-based TO procedures, thereby maximising its implementation fidelity in the broader community.

## Electronic supplementary material

Below is the link to the electronic supplementary material.

Supplementary Material 1

Supplementary Material 2

## Data Availability

The data that support the findings of this study are available from the corresponding author upon reasonable request.
