# Supplementary material for: Generation time-out grows up: young adults’ reports about childhood time-out use and their mental health, attachment, and emotion regulation
Source: Eur Child Adolesc Psychiatry. 2024 Mar 5;33(10):3471–9. doi: 10.1007/s00787-024-02408-8 (PMC11564331; doi:10.1007/s00787-024-02408-8)
Supplement: Supplementary file 1 — Supplementary Material 1 [file 787_2024_2408_MOESM1_ESM.pdf]

Generation Time-out Grows Up: Young Adults' Reports About Childhood Time-out Use and Their Mental Health, Attachment, and Emotion Regulation

European Child & Adolescent Psychiatry

Jingyi Xu, BPsych (Hons); Lucy A. Tully, PhD; Mark R. Dadds, PhD

The University of Sydney, Sydney, Australia

Supplementary Material

Corresponding author

Mark R. Dadds, The University of Sydney, Camperdown, Sydney, AU-NSW 2008, Australia.

Email: mark.dadds@sydney.edu.au

Online Resource Material

1. eTable 1. Evidence-based Time-out Parameters and Corresponding Scale Items
2. eMethods. Supplemental Methods
3. eTable 2. Perceived Impacts of Childhood Use of Time-out and Other Discipline on Current Mental Health Outcomes
4. eResults. Supplemental Results (Study 2)
5. References

Online Resource 1

eTable 1. Evidence-based Time-out Parameters and Corresponding Scale Items[a]

Evidence-based Parameter	Item	Response Range	
		Least evidence-based	More evidence-based
TO use on operant child behaviours	1. Did your parent(s)/caregiver(s) use time-out when you made a mistake or did something bad by accident as a child?	Always	Never
	2. Did your parent(s)/caregiver(s) use time-out when you refused to do something due to fear and anxiety as a child?	Always	Never
Consistent implementation	3. When your parent(s)/caregiver(s) used time-out, they would tend to:	Vary the way they use it every time	Use it the same way every time
Immediate TO use contingent on misbehaviour	4. When you misbehaved as a child, your parent(s)/caregiver(s) tended to:	Wait as long as possible before using time-out	Intervene with time-out quickly
Calm delivery of TO	5. When putting you in time-out as a child, your parent(s)/caregiver(s) acted:	Angry and distressed	Calm and quiet
Appropriate TO location with minimal reinforcement	6. The room or space that you usually gone to for time out:	Involved activities, books and/or things for you to do	Was always boring, with no activities
Minimal parental attention during TO	7. When you were in time-out as a child, your parent(s)/caregiver(s) would typically:	Talk to you and respond to questions	Ignore you
Enforcing a back-up consequence to TO escape	8. If you left time-out without permission as a child, your parent(s)/caregiver(s) would:	Let it go and move on	Always have a back-up consequence (e.g., go back to time-out, lose screen time)
Parent monitored TO release	9. When you were in time-out as a child:	You could decide when to end time-out	Your parent(s)/ caregiver(s) would decide when you were allowed out of time-out
Contingent release on calm behaviour	10. When you left time-out as a child, you were usually:	Angry	Calm
Parent-child reconciliation after TO	11. After timeout had finished, your parent(s)/caregiver(s) would tend to:	Stay upset with you for some time	Move on quickly
Restatement of the original command	12. If using time-out for you not following instructions as a child, your parent(s)/caregiver(s) would:	Forget about the instruction and move on	Always make you follow the instruction after time-out

[a] TO = time-out. Responses were rated on a 5-point Likert scale.

Online Resource 2

eMethods. Supplementary Methods

Measures

Adult Attachment Scale (AAS)

Young adults' current secure attachment was measured using the AAS [1]. This measure was skipped for participants who indicated that they had never been in a romantic relationship. Participants responded on a 5-point scale ranging from *Not at all True* to *Very True*. Scores were computed as the item sum scores, with higher scores indicative of more insecure attachment. The scale has demonstrated acceptable internal consistency [2, 3].

Difficulties in Emotion Regulation Scale (DERS)

The DERS was used as a measure of emotion dysregulation [4]. Participants indicated the extent to which each statement corresponds with their reactions to negative emotions on a 5-point scale ranging from *Almost Never* to *Almost Always*. Higher sum scores indicate greater emotion dysregulation. The DERS has shown good internal consistency [4, 5].

K10

The K10 is a mental health screener that measures psychological distress based on the DSM-IV [6]. Participants responded on their current mental well-being on a 5-point scale ranging from *None of the Time* to *All of the Time*. Higher sum scores represent more psychological distress. The K10 is internationally validated, demonstrating strong psychometric properties of high internal consistency [6].

Descriptions of Parental Caregiving Style (DPCS)

Childhood parental attachment was assessed through the DPCS [7]. The DPCS is a retrospective measure containing three descriptions that characterise the parental attachment styles of secure, anxious, and ambivalent. Only the secure attachment item was used. Participants rated the extent to which the description resembled each caregiver's caregiving attitudes and behaviours in childhood, using a 9-point scale ranging from *Not at all True* to *Extremely True*. Higher scores indicate more secure attachment. The scale has demonstrated adequate internal consistency [8].

Parenting Styles and Dimensions Questionnaire – Intergenerational Version (PSDQ-G1)

The PSDQ-G1 was used to measure adults' perception of parenting received in childhood [9]. Of the three subscales based on Baumrind's [10] typology of parenting styles, this study utilised the authoritative and authoritarian parenting subscales. Items in each subscale reflect specific parenting practices that occur within the context of each parenting style. Participants reported the frequencies in which each identified caregiver(s) engaged in each statement on a 5-point scale ranging from *Never* to *Always*. Scores were computed as the mean score of items within each subscale, where higher scores indicate greater attributes on the parenting style. The scale has shown good internal consistency across subscales [11].

Adverse Life Experience Scale (ALES)

The ALES was used to assess individuals' exposure to adversity in life [12]. Items consisted of binary responses concerning the occurrence of a range of life adversities and follow-up questions regarding the developmental period at which the adversity occurred, ranging from *0–1 Year* to *Adulthood*. Participants indicated all developmental timings that applied. Exposure to childhood adversity scores was calculated as the total adversities reported between the age of 0–8 years. The ALES has shown good validity and internal consistency [12].

Online Resource 3

eTable 2. Perceived Impacts of Childhood Use of Time-out and Other Discipline on Current Mental Health Outcomes

Discipline type	Negative Impact		No impact		Positive Impact	
	N	%	N	%	N	%
**Childhood use of time-out (n = 334)**						
Current attachment style	76	22·8	208	62·2	50	15·0
Current emotion regulation	65	19·5	133	39·8	136	40·7
Current mental health	83	24·9	190	56·8	61	18·3
**Childhood use of any other discipline (N = 407)**						
Current attachment style	142	34·9	147	36·1	118	29·0
Current emotion regulation	172	42·3	64	15·7	171	42·0
Current mental health	191	46·9	102	25·1	114	28·0

Online Resource 4

eResults. Supplementary Results (Study 2)

As hypothesised, time-out (TO) was a commonly used discipline strategy with 465 young adults (86·9%) reporting experiencing time-out in childhood, and 137 (25·6%) indicated TO is a frequently or always used strategy. As with the findings in Study 1, the degree of adherence to evidence-based parameters of TO use was widely varied (eTable 3).

Perceptions of TO

While 204 young adults (38·1%) reported TO as an effective discipline technique, 192 (35·9%) considered TO as neither effective nor ineffective and 139 (26%) endorsed TO as ineffective. Replicating the findings in Study 1, TO was regarded as the second most acceptable technique after taking away a privilege, followed by yelling, smacking and shaming (eTable 4). Overall, TO was perceived as having more positive impacts on young adults' mental health outcomes than other types of discipline used in childhood (eTable 5). Further, more appropriate implementation of TO was positively associated with greater perceived TO effectiveness (r=0·28 [95% CI 0·19–0·36], p<0.01), acceptability (r=0·25 [95% CI 0·17–0·34], p<0·01), and positive impacts on adulthood outcomes (r=0·23 [95% CI 0·14–0·32], p<0·01).

eTable 3. Percentages of Adherence to the Evidence-based Parameters of Time-out in Study 2[a]

Evidence-based parameter	n	%
TO use on operant child behaviours[b]	268	57·6
Consistent implementation	242	52·0
Immediate TO use contingent on misbehaviour	145	31·1
Calm delivery of TO	124	26·7
Appropriate TO location with minimal reinforcement	251	54·0
Minimal parental attention during TO	225	48·3
Enforcing a back-up consequence to TO escape	297	63·9
Parent monitored TO release	327	70·3
Contingent release on calm behaviour	182	39·1
Parent-child reconciliation after TO	163	35·1
Restatement of the original command	257	55·2

[a] TO = time-out.
[b] Values were calculated as the average of the items on the parameter.

eTable 4. Descriptive Statistics for Discipline Acceptability Ratings in Study 2[a]

Discipline technique	Mean	SD	95% CI
Take away a privilege	3·29[b]	1·23	3·18–3·39
Time-out	3·09[b]	1·20	2·99–3·20
Yelling	2·15[b]	1·04	2·07–2·24
Smacking	1·73[b]	0·98	1·64–1·81
Shaming	1·60[b]	0·92	1·52–1·68

[a] Acceptability scores were rated on a 5-point scale ranging from *Not at all acceptable* to *Very acceptable*.
[b] Means with the same superscript are significantly different from each other at $p<0·01$.

eTable 5. Perceived Impacts of Childhood Use of Time-out and Other Discipline on Current Mental Health Outcomes in Study 2

Discipline type	Negative Impact		No impact		Positive Impact	
	N	%	N	%	N	%
Childhood use of time-out (n = 465)						
Current attachment style	118	25·4	255	54·8	92	19·8
Current emotion regulation	127	27·3	196	42·2	142	30·5
Current mental health	135	29·0	219	47·1	111	23·9
Childhood use of any other discipline (N = 535)						
Current attachment style	177	33·1	240	44·9	118	22·1
Current emotion regulation	194	36·3	196	36·6	145	27·1
Current mental health	192	35·9	231	43·2	112	20·9

References

1. Collins NL, Read SJ (1990) Adult attachment, working models, and relationship quality in dating couples. Journal of personality and social psychology 58:644. https://doi.org/10.1037/0022-3514.58.4.644
2. Collins NL (1996) Working models of attachment: Implications for explanation, emotion, and behavior. Journal of personality and social psychology 71:810-832. https://doi.org/10.1037/0022-3514.71.4.810
3. Ravitz P, Maunder R, Hunter J, Sthankiya B, Lancee W (2010) Adult attachment measures: A 25-year review. Journal of psychosomatic research 69:419-432. https://doi.org/10.1016/j.jpsychores.2009.08.006
4. Gratz KL, Roemer L (2004) Multidimensional assessment of emotion regulation and dysregulation: Development, factor structure, and initial validation of the difficulties in emotion regulation scale. Journal of psychopathology and behavioral assessment 26:41-54. https://doi.org/10.1023/B:JOBA.0000007455.08539.94
5. Gratz KL, Paulson A, Jakupcak M, Tull MT (2009) Exploring the relationship between childhood maltreatment and intimate partner abuse: Gender differences in the mediating role of emotion dysregulation. Violence and Victims 24:68-82. https://doi.org/10.1891/0886-6708.24.1.68
6. Kessler RC, Andrews G, Colpe LJ, Hiripi E, Mroczek DK, Normand S-L, et al (2002) Short screening scales to monitor population prevalences and trends in non-specific psychological distress. Psychological medicine 32:959-976. https://doi.org/10.1017/S0033291702006074
7. Smallbone SW, Dadds MR (1998) Childhood attachment and adult attachment in incarcerated adult male sex offenders. Journal of interpersonal violence 13:555-573. https://doi.org/10.1177/088626098013005001

8. Dalton III WT, Frick-Horbury D, Kitzmann KM (2006) Young adults' retrospective reports of parenting by mothers and fathers: Associations with current relationship quality. The Journal of general psychology 133:5-18. https://doi.org/10.3200/GENP.133.1.5-18

9. Robinson CC, Mandleco B, Olsen SF, Hart CH (2001) The parenting styles and dimensions questionnaire (PSDQ). Handbook of family measurement techniques 3:319-321.

10. Baumrind D (1971) Current patterns of parental authority. Developmental psychology 4(1):1-103. https://doi.org/10.1037/h0030372

11. Tagliabue S, Olivari MG, Bacchini D, Affuso G, Confalonieri E (2014) Measuring adolescents' perceptions of parenting style during childhood: psychometric properties of the parenting styles and dimensions questionnaire. Psicologia: Teoria e Pesquisa 30:251-258. https://doi.org/10.1590/S0102-37722014000300002

12. Hawes DJ, Lechowicz M, Roach A, Fisher C, Doyle FL, Noble S, et al (2021) Capturing the developmental timing of adverse childhood experiences: The Adverse Life Experiences Scale. American Psychologist 76:253-267. https://doi.org/10.1037/amp0000760
