# Supplementary material for: Generation time-out grows up: young adults’ reports about childhood time-out use and their mental health, attachment, and emotion regulation
Source: Eur Child Adolesc Psychiatry. 2024 Mar 5;33(10):3471–9. doi: 10.1007/s00787-024-02408-8 (PMC11564331; doi:10.1007/s00787-024-02408-8)
Supplement: Supplementary file 2 — Supplementary Material 2 [file 787_2024_2408_MOESM2_ESM.docx]

**Generation Time-out Grows Up: Young Adults’ Reports About Childhood Time-out Use and Their Mental Health, Attachment, and Emotion Regulation**

European Child & Adolescent Psychiatry

Jingyi Xu, BPsych (Hons); Lucy A. Tully, PhD; Mark R. Dadds, PhD

The University of Sydney, Sydney, Australia

Supplementary Material

**Corresponding author**

Mark R. Dadds, The University of Sydney, Camperdown, Sydney, AU-NSW 2008, Australia. Email: [mark.dadds@sydney.edu.au](mailto:mark.dadds@sydney.edu.au)

**Online Resource Material**

1. eTable 1. Evidence-based Time-out Parameters and Corresponding Scale Items
2. eMethods. Supplemental Methods
3. eResults. Supplemental Results (Study 1)
4. eResults. Supplemental Results (Study 2)
5. References

**Online Resource 1**

**eTable 1.** Evidence-based Time-out Parameters and Corresponding Scale Items^a^

| Evidence-based Parameter | Item | Response Range | |
| --- | --- | --- | --- |
|  |  | Least evidence-based | More evidence-based |
| TO use on operant child behaviours | 1. Did your parent(s)/caregiver(s) use time-out when you made a mistake or did something bad by accident as a child? | Always | Never |
|  | 1. Did your parent(s)/caregiver(s) use time-out when you refused to do something due to fear and anxiety as a child? | Always | Never |
| Consistent implementation | 1. When your parent(s)/caregiver(s) used time-out, they would tend to: | Vary the way they use it every time | Use it the same way every time |
| Immediate TO use contingent on misbehaviour | 1. When you misbehaved as a child, your parent(s)/caregiver(s) tended to: | Wait as long as possible before using time-out | Intervene with time-out quickly |
| Calm delivery of TO | 1. When putting you in time-out as a child, your parent(s)/caregiver(s) acted: | Angry and distressed | Calm and quiet |
| Appropriate TO location with minimal reinforcement | 1. The room or space that you usually gone to for time out: | Involved activities, books and/or things for you to do | Was always boring, with no activities |
| Minimal parental attention during TO | 1. When you were in time-out as a child, your parent(s)/caregiver(s) would typically: | Talk to you and respond to questions | Ignore you |
| Enforcing a back-up consequence to TO escape | 1. If you left time-out without permission as a child, your parent(s)/caregiver(s) would: | Let it go and move on | Always have a back-up consequence (e.g., go back to time-out, lose screen time) |
| Parent monitored TO release | 1. When you were in time-out as a child: | You could decide when to end time-out | Your parent(s)/ caregiver(s) would decide when you were allowed out of time-out |
| Contingent release on calm behaviour | 1. When you left time-out as a child, you were usually: | Angry | Calm |
| Parent-child reconciliation after TO | 1. After timeout had finished, your parent(s)/caregiver(s) would tend to: | Stay upset with you for some time | Move on quickly |
| Restatement of the original command | 1. If using time-out for you not following instructions as a child, your parent(s)/caregiver(s) would: | Forget about the instruction and move on | Always make you follow the instruction after time-out |

^a^ TO = time-out. Responses were rated on a 5-point Likert scale.

**Online Resource 2**

**eMethods. Supplementary Methods**

### Measures

*Note*. Means, SDs and Cronbach’s alphas reported below are based on Study 1 only as results are similar across the two studies.

### Adult Attachment Scale (AAS)

Young adults’ current secure attachment was measured using the AAS [1]. This measure was skipped for participants who indicated that they had never been in a romantic relationship. Participants responded on a 5-point scale ranging from *Not at all True* to *Very True*. Two subscale scores (anxiety and avoid) were computed with higher scores indicative of more anxious/avoidant attachment. The mean scores for the anxiety and avoid subscale were 13.67 (SD = 5.29) and 32.10 (*SD* = 9.58), respectively. The subscales have demonstrated good internal consistency in past research [2, 3] and in the current study (Cronbach’s alpha = 0.78-0.84).

###

### Difficulties in Emotion Regulation Scale (DERS)

The DERS was used as a measure of emotion dysregulation [4]. Participants indicated the extent to which each statement corresponds with their reactions to negative emotions on a 5-point scale ranging from *Almost Never* to *Almost Always*. Higher sum scores indicate greater emotion dysregulation. The DERS had a mean score of 94.80 (*SD* = 24.95). As with previous research [4, 5], it also exhibited good internal consistency (Cronbach’s alpha = 0.94) in this study.

###

### K10

The K10 is a mental health screener that measures psychological distress based on the DSM-IV [6]. Participants responded on their current mental well-being on a 5-point scale ranging from *None of the Time* to *All of the Time*. Higher sum scores represent more psychological distress. The K10 is internationally validated, demonstrating strong psychometric properties of high internal consistency [6]. Mean score of the K10 in this study was 24.06 (*SD* = 7.52). The Cronbach’s alpha was 0.90.

###

### Descriptions of Parental Caregiving Style (DPCS)

Childhood parental attachment was assessed through the DPCS [7]. The DPCS is a retrospective measure containing three descriptions that characterise the parental attachment styles of secure, anxious, and ambivalent. Only the secure attachment item was used. Participants rated the extent to which the description resembled each caregiver’s caregiving attitudes and behaviours in childhood, using a 9-point scale ranging from *Not at all True* to *Extremely True*. Higher scores indicate more secure attachment. Mean DPCS score was 6.78 (SD = 2.31) in this study. While this study did not examine the internal consistency as only one item was use, the scale has demonstrated adequate internal consistency [8].

###

### Parenting Styles and Dimensions Questionnaire – Intergenerational Version (PSDQ-G1)

The PSDQ-G1 was used to measure adults’ perception of parenting received in childhood [9]. Of the three subscales based on Baumrind’s [10] typology of parenting styles, this study utilised the authoritative and authoritarian parenting subscales. Items in each subscale reflect specific parenting practices that occur within the context of each parenting style. Participants reported the frequencies in which each identified caregiver(s) engaged in each statement on a 5-point scale ranging from *Never* to *Always*. Scores were computed as the mean score of items within each subscale, where higher scores indicate greater attributes on the parenting style. Mean scores on the authoritarian and authoritative subscale were 2.40 (*SD* = 0.85) and 3.18 (*SD* = 0.89) with the Cronbach’s alphas of 0.91 and 0.94, respectively. The scale has shown good internal consistency across subscales [11].

###

### Adverse Life Experience Scale (ALES)

The ALES was used to assess individuals’ exposure to adversity in life [12]. Items consisted of binary responses concerning the occurrence of a range of life adversities and follow-up questions regarding the developmental period at which the adversity occurred, ranging from *0–1 Year* to *Adulthood*. Participants indicated all developmental timings that applied. Exposure to childhood adversity scores was calculated as the total adversities reported between the age of 0–8 years (*M* = 2.58, *SD* = 4.00). The ALES has shown good validity and internal consistency [12]. The Cronbach’s alpha in this study was 0.75.

**Online Resource 3**

**eResults. Supplementary Results (Study 1)**

**eTable 2.** Perceived Impacts of Childhood Use of Time-out and Other Discipline on Current Mental Health Outcomes in Study 1

| Discipline type | Negative Impact | | No impact | | Positive Impact | |
| --- | --- | --- | --- | --- | --- | --- |
|  | **N** | **%** | **N** | **%** | **N** | **%** |
| Childhood use of time-out (n = 334) | | | | | | |
| Current attachment style | 76 | 22·8 | 208 | 62·2 | 50 | 15·0 |
| Current emotion regulation | 65 | 19·5 | 133 | 39·8 | 136 | 40·7 |
| Current mental health | 83 | 24·9 | 190 | 56·8 | 61 | 18·3 |
| Childhood use of any other discipline (N = 407) | | | | | | |
| Current attachment style | 142 | 34·9 | 147 | 36·1 | 118 | 29·0 |
| Current emotion regulation | 172 | 42·3 | 64 | 15·7 | 171 | 42·0 |
| Current mental health | 191 | 46·9 | 102 | 25·1 | 114 | 28·0 |

**eTable 3.** Full results - adjusted Associations between Appropriateness of Time-out Implementation and Childhood Adversity with Insecure Attachment, Emotion Dysregulation and Mental Health Problems in Study 1

| Dependent variable | Independent variable | B | SE | 95% CI | *p* | *sr ^2^* ^b^ |
| --- | --- | --- | --- | --- | --- | --- |
| Avoidant attachment | *Step 1 (R^2^=.212)* |  |  |  |  |  |
|  | Childhood adversity | 0.15 | 0.20 | -0.24 to 0.54 | 0.457 | .00 |
|  | Adulthood adversity | 0.04 | 0.13 | -0.21 to 0.29 | 0.733 | .00 |
|  | Secure childhood attachment | -0.45 | 0.39 | -1.20 to 0.29 | 0.233 | .01 |
|  | Authoritative parenting | -2.22 | 1.04 | -4.27 to -0.16 | 0.035 | .02 |
|  | Authoritarian parenting | 0.18 | 0.82 | -1.44 to 1.80 | 0.826 | .00 |
|  | Appropriate TO implementation | -0.32 | 0.11 | -0.54 to -0.10 | 0.004 | .04 |
|  | *Step 2 (R^2^=.212)* |  |  |  |  |  |
|  | Childhood adversity | 0.15 | 0.20 | -0.25 to 0.54 | 0.462 | .00 |
|  | Adulthood adversity | 0.04 | 0.13 | -0.21 to 0.29 | 0.735 | .00 |
|  | Secure childhood attachment | -0.45 | 0.38 | -1.20 to 0.30 | 0.241 | .01 |
|  | Authoritative parenting | -2.22 | 1.05 | -4.28 to -0.16 | 0.035 | .02 |
|  | Authoritarian parenting | 0.19 | 0.83 | -1.45 to 1.82 | 0.822 | .00 |
|  | Appropriate TO implementation | -0.32 | 0.11 | -0.54 to -0.10 | 0.004 | .04 |
|  | Childhood adversity by Appropriate TO implementation | -0.002 | 0.03 | -0.06 to 0.06 | 0.945 | .00 |
| Anxious attachment | *Step 1 (R^2^=.133)* |  |  |  |  |  |
|  | Childhood adversity | 0.11 | 0.13 | -0.15 to 0.36 | 0.413 | .00 |
|  | Adulthood adversity | 0.06 | 0.08 | -0.10 to 0.22 | 0.477 | .00 |
|  | Secure childhood attachment | 0.36 | 0.25 | -0.14 to 0.83 | 0.162 | .01 |
|  | Authoritative parenting | -1.38 | 0.68 | -2.72 to -0.06 | 0.043 | .02 |
|  | Authoritarian parenting | 0.95 | 0.53 | -0.11 to 2.00 | 0.078 | .02 |
|  | Appropriate TO implementation | -0.15 | 0.72 | -0.29 to -0.01 | 0.034 | .03 |
|  | *Step 2 (R^2^=.136)* |  |  |  |  |  |
|  | Childhood adversity | 0.11 | 0.13 | -0.14 to 0.37 | 0.380 | .00 |
|  | Adulthood adversity | 0.06 | 0.08 | -0.10 to 0.22 | 0.466 | .00 |
|  | Secure childhood attachment | 0.32 | 0.25 | -0.17 to 0.81 | 0.198 | .01 |
|  | Authoritative parenting | -1.37 | 0.68 | -2.71 to -0.03 | 0.046 | .02 |
|  | Authoritarian parenting | 0.89 | 0.54 | -0.17 to 1.96 | 0.098 | .02 |
|  | Appropriate TO implementation | -0.15 | 0.07 | -0.29 to -0.01 | 0.035 | .03 |
|  | Childhood adversity by Appropriate TO implementation | 0.015 | 0.18 | -0.02 to 0.05 | 0.400 | .00 |
| Emotion dysregulation | *Step 1 (R^2^=.250)* |  |  |  |  |  |
|  | Childhood adversity | 1.25 | 0.43 | 0.41 to 2.09 | 0.004 | .02 |
|  | Adulthood adversity | 0.44 | 0.28 | -0.11 to 0.99 | 0.109 | .01 |
|  | Secure childhood attachment | 1.15 | 0.78 | -0.39 to 2.69 | 0.143 | .01 |
|  | Authoritative parenting | 3.39 | 1.75 | -0.06 to 6.84 | 0.001 | .03 |
|  | Authoritarian parenting | -6.65 | 2.07 | -10.73 to -2.57 | 0.053 | .01 |
|  | Appropriate TO implementation | -0.81 | 0.22 | -1.24 to -0.37 | <0.001 | .03 |
|  | *Step 2 (R^2^=.250)* |  |  |  |  |  |
|  | Childhood adversity | 1.25 | 0.43 | 0.41 to 2.10 | 0.004 | .02 |
|  | Adulthood adversity | 0.44 | 0.28 | -0.11 to 0.99 | 0.109 | .01 |
|  | Secure childhood attachment | 1.15 | 0.78 | -0.39 to 2.69 | 0.143 | .01 |
|  | Authoritative parenting | -6.65 | 2.07 | -10.73 to -2.57 | 0.001 | .03 |
|  | Authoritarian parenting | 3.39 | 1.75 | -0.06 to 6.84 | 0.053 | .01 |
|  | Appropriate TO implementation | -0.81 | 0.22 | -1.25 to -0.37 | <0.001 | .03 |
|  | Childhood adversity by Appropriate TO implementation | 0.002 | 0.06 | -0.12 to 0.12 | 0.979 | .00 |
| Mental health problems | *Step 1 (R^2^=.212)* |  |  |  |  |  |
|  | Childhood adversity | 0.26 | 0.14 | -0.02 to 0.53 | 0.064 | .01 |
|  | Adulthood adversity | 0.29 | 0.09 | 0.11 to 0.47 | 0.001 | .03 |
|  | Secure childhood attachment | 0.13 | 0.25 | -0.36 to 0.62 | 0.623 | .00 |
|  | Authoritative parenting | -0.22 | 0.67 | -1.54 to 1.10 | 0.744 | .00 |
|  | Authoritarian parenting | 1.39 | 0.57 | 0.26 to 2.51 | 0.015 | .02 |
|  | Appropriate TO implementation | -0.24 | 0.07 | -0.38 to -0.10 | 0.001 | .03 |
|  | *Step 2 (R^2^=.216)* |  |  |  |  |  |
|  | Childhood adversity | 0.27 | 0.14 | -0.01 to 0.54 | 0.059 | .01 |
|  | Adulthood adversity | 0.30 | 0.09 | 0.12 to 0.48 | 0.001 | .03 |
|  | Secure childhood attachment | 0.11 | 0.26 | -0.40 to 0.62 | 0.676 | .00 |
|  | Authoritative parenting | -0.17 | 0.67 | -1.49 to 1.15 | 0.807 | .00 |
|  | Authoritarian parenting | 1.40 | 0.57 | 0.28 to 2.52 | 0.015 | .02 |
|  | Appropriate TO implementation | -0.23 | 0.07 | -0.38 to -0.09 | 0.002 | .03 |
|  | Childhood adversity by Appropriate TO implementation | 0.02 | 0.02 | -0.02 to 0.06 | 0.241 | .00 |

^a^ Squared semi-partial correlation, representing the unique variance accounted for by each variable.

**eResults. Supplementary Results (Study 2)**

As hypothesised, time-out (TO) was a commonly used discipline strategy with 465 young adults (86·9%) reporting experiencing time-out in childhood, and 137 (25·6%) indicated TO is a frequently or always used strategy. As with the findings in Study 1, the degree of adherence to evidence-based parameters of TO use was widely varied (eTable 4).

**Perceptions of TO**

While 204 young adults (38·1%) reported TO as an effective discipline technique, 192 (35·9%) considered TO as neither effective nor ineffective and 139 (26%) endorsed TO as ineffective. Replicating the findings in Study 1, TO was regarded as the second most acceptable technique after taking away a privilege, followed by yelling, smacking and shaming (eTable 5). Overall, TO was perceived as having more positive impacts on young adults’ mental health outcomes than other types of discipline used in childhood (eTable 6). Further, more appropriate implementation of TO was positively associated with greater perceived TO effectiveness (*r*=0·28 [95% CI 0·19–0·36], p<0.01), acceptability (*r*=0·25 [95% CI 0·17–0·34], p<0·01), and positive impacts on adulthood outcomes (*r*=0·23 [95% CI 0·14–0·32], p<0·01).

**eTable 4.** Percentages of Adherence to the Evidence-based Parameters of Time-out in Study 2^a^

| Evidence-based parameter | n | % |
| --- | --- | --- |
| TO use on operant child behaviours^b^ | 268 | 57·6 |
| Consistent implementation | 242 | 52·0 |
| Immediate TO use contingent on misbehaviour | 145 | 31·1 |
| Calm delivery of TO | 124 | 26·7 |
| Appropriate TO location with minimal reinforcement | 251 | 54·0 |
| Minimal parental attention during TO | 225 | 48·3 |
| Enforcing a back-up consequence to TO escape | 297 | 63·9 |
| Parent monitored TO release | 327 | 70·3 |
| Contingent release on calm behaviour | 182 | 39·1 |
| Parent-child reconciliation after TO | 163 | 35·1 |
| Restatement of the original command | 257 | 55·2 |

^a^ TO = time-out.

^b^ Values were calculated as the average of the items on the parameter.

**eTable 5.** Descriptive Statistics for Discipline Acceptability Ratings in Study 2^a^

| Discipline technique | Mean | SD | 95% CI |
| --- | --- | --- | --- |
| Take away a privilege | 3·29^b^ | 1·23 | 3·18–3·39 |
| Time-out | 3·09^b^ | 1·20 | 2·99–3·20 |
| Yelling | 2·15^b^ | 1·04 | 2·07–2·24 |
| Smacking | 1·73^b^ | 0·98 | 1·64–1·81 |
| Shaming | 1·60^b^ | 0·92 | 1·52–1·68 |

^a^ Acceptability scores were rated on a 5-point scale ranging from *Not at all acceptable* to *Very acceptable*.

^b^ Means with the same superscript are significantly different from each other at p<0·01.

**eTable 6.** Perceived Impacts of Childhood Use of Time-out and Other Discipline on Current Mental Health Outcomes in Study 2

| Discipline type | Negative Impact | | No impact | | Positive Impact | |
| --- | --- | --- | --- | --- | --- | --- |
|  | N | % | N | % | N | % |
| Childhood use of time-out (n = 465) |  |  |  |  |  |  |
| Current attachment style | 118 | 25·4 | 255 | 54·8 | 92 | 19·8 |
| Current emotion regulation | 127 | 27·3 | 196 | 42·2 | 142 | 30·5 |
| Current mental health | 135 | 29·0 | 219 | 47·1 | 111 | 23·9 |
| Childhood use of any other discipline (N = 535) | | | | | | |
| Current attachment style | 177 | 33·1 | 240 | 44·9 | 118 | 22·1 |
| Current emotion regulation | 194 | 36·3 | 196 | 36·6 | 145 | 27·1 |
| Current mental health | 192 | 35·9 | 231 | 43·2 | 112 | 20·9 |

**eTable 7**. Full results - Adjusted Associations between Appropriateness of Time-out Implementation and Childhood Adversity with Insecure Attachment, Emotion Dysregulation and Mental Health Problems in Study 2

| Dependent variable | Independent variable | B | SE | 95% CI | *p* | *sr^2^* ^a^ |
| --- | --- | --- | --- | --- | --- | --- |
| Avoidant attachment | *Step 1 (R^2^=.206)* |  |  |  |  |  |
|  | Childhood adversity | 0.01 | 0.07 | -0.12 to 0.13 | 0.914 | .00 |
|  | Adulthood adversity | 0.27 | 0.05 | 0.16 to 0.36 | <.001 | .07 |
|  | Secure childhood attachment | -0.24 | 0.20 | -0.63 to 0.16 | 0.241 | .00 |
|  | Authoritative parenting | -0.69 | 0.55 | -1.78 to 0.40 | 0.215 | .00 |
|  | Authoritarian parenting | 1.27 | 0.52 | 0.25 to 2.28 | 0.015 | .02 |
|  | Appropriate TO implementation | -0.01 | 0.07 | -0.15 to 0.14 | 0.921 | .00 |
|  | *Step 2 (R^2^=.206)* |  |  |  |  |  |
|  | Childhood adversity | -0.002 | 0.07 | -0.13 to 0.13 | 0.974 | .00 |
|  | Adulthood adversity | 0.26 | 0.05 | 0.16 to 0.36 | <.001 | .07 |
|  | Secure childhood attachment | -0.23 | 0.20 | -0.63 to 0.16 | 0.248 | .00 |
|  | Authoritative parenting | -0.68 | 0.55 | -1.77 to 0.41 | 0.219 | .00 |
|  | Authoritarian parenting | 1.28 | 0.52 | 0.26 to 2.30 | 0.014 | .02 |
|  | Appropriate TO implementation | -0.01 | 0.07 | -0.15 to 0.14 | 0.938 | .00 |
|  | Childhood adversity by Appropriate TO implementation | -0.005 | 0.01 | -0.02 to 0.01 | 0.570 | .00 |
| Anxious attachment | *Step 1 (R^2^=.218)* |  |  |  |  |  |
|  | Childhood adversity | 0.01 | 0.04 | -0.08 to 0.10 | 0.788 | .00 |
|  | Adulthood adversity | 0.17 | 0.03 | 0.10 to 0.23 | <.001 | .07 |
|  | Secure childhood attachment | 0.03 | 0.13 | -0.24 to 0.29 | 0.837 | .00 |
|  | Authoritative parenting | 0.64 | 0.37 | -0.09 to 1.36 | 0.085 | .01 |
|  | Authoritarian parenting | 1.55 | 0.34 | 0.88 to 2.23 | <.001 | .06 |
|  | Appropriate TO implementation | -0.12 | 0.05 | -0.22 to -0.02 | 0.017 | .02 |
|  | *Step 2 (R^2^=.218)* |  |  |  |  |  |
|  | Childhood adversity | 0.01 | 0.04 | -0.08 to 0.10 | 0.769 | .00 |
|  | Adulthood adversity | 0.17 | 0.03 | 0.10 to 0.23 | <.001 | .07 |
|  | Secure childhood attachment | 0.03 | 0.13 | -0.24 to 0.29 | 0.841 | .00 |
|  | Authoritative parenting | 0.63 | 0.37 | -0.10 to 1.36 | 0.086 | .01 |
|  | Authoritarian parenting | 1.55 | 0.34 | 0.87 to 2.23 | <.001 | .06 |
|  | Appropriate TO implementation | -0.12 | 0.05 | -0.22 to -0.02 | 0.017 | .02 |
|  | Childhood adversity by Appropriate TO implementation | 0.001 | 0.01 | -0.01 to 0.01 | 0.890 | .00 |
| Emotion dysregulation | *Step 1 (R^2^=.246)* |  |  |  |  |  |
|  | Childhood adversity | 0.33 | 0.18 | -0.02 to 0.68 | 0.064 | .01 |
|  | Adulthood adversity | 0.67 | 0.14 | 0.40 to 0.93 | <.001 | .04 |
|  | Secure childhood attachment | -0.11 | 0.57 | -1.22 to 1.01 | 0.850 | .00 |
|  | Authoritative parenting | 0.82 | 1.55 | -2.24 to 3.87 | 0.599 | .00 |
|  | Authoritarian parenting | 8.03 | 1.14 | 5.26 to 10.80 | <.001 | .06 |
|  | Appropriate TO implementation | -0.21 | 0.20 | -0.60 to 0.20 | 0.314 | .00 |
|  | *Step 2 (R^2^=.253)* |  |  |  |  |  |
|  | Childhood adversity | 0.42 | 0.18 | 0.06 to 0.78 | 0.022 | .01 |
|  | Adulthood adversity | 0.66 | 0.14 | 0.39 to 0.93 | <.001 | .04 |
|  | Secure childhood attachment | -0.15 | 0.57 | -1.27 to 0.96 | 0.785 | .00 |
|  | Authoritative parenting | 0.80 | 1.55 | -2.24 to 3.84 | 0.606 | .00 |
|  | Authoritarian parenting | 7.89 | 1.41 | 5.12 to 10.66 | <.001 | .06 |
|  | Appropriate TO implementation | -0.19 | 0.20 | -0.59 to 0.21 | 0.344 | .00 |
|  | Childhood adversity by Appropriate TO implementation | 0.05 | 0.03 | 0.002 to 0.10 | 0.042 | .01 |
| Mental health problems | *Step 1 (R^2^=.238)* |  |  |  |  |  |
|  | Childhood adversity | 0.10 | 0.07 | -0.03 to 0.23 | 0.124 | .01 |
|  | Adulthood adversity | 0.33 | 0.05 | 0.23 to 0.43 | <.001 | .10 |
|  | Secure childhood attachment | -0.06 | 0.21 | -0.46 to 0.35 | 0.776 | .00 |
|  | Authoritative parenting | 1.20 | 0.57 | 0.09 to 2.31 | 0.035 | .01 |
|  | Authoritarian parenting | 1.71 | 0.51 | 0.70 to 2.72 | 0.001 | .03 |
|  | Appropriate TO implementation | -0.22 | 0.07 | -0.37 to -0.08 | 0.003 | .02 |
|  | *Step 2 (R^2^=.239)* |  |  |  |  |  |
|  | Childhood adversity | 0.11 | 0.07 | -0.02 to 0.25 | 0.085 | .01 |
|  | Adulthood adversity | 0.33 | 0.05 | 0.23 to 0.43 | <.001 | .10 |
|  | Secure childhood attachment | -0.07 | 0.21 | -0.47 to 0.34 | 0.747 | .00 |
|  | Authoritative parenting | 1.19 | 0.57 | 0.08 to 2.31 | 0.035 | .01 |
|  | Authoritarian parenting | 1.69 | 0.51 | 0.68 to 2.70 | 0.001 | .03 |
|  | Appropriate TO implementation | -0.22 | 0.07 | -0.37 to -0.07 | 0.003 | .02 |
|  | Childhood adversity by Appropriate TO implementation | 0.01 | 0.01 | -0.01 to 0.03 | 0.340 | .00 |

^a^ Squared semi-partial correlation, representing the unique variance accounted for by each variable.

**References**

1. Collins NL, Read SJ (1990) Adult attachment, working models, and relationship quality in dating couples. Journal of personality and social psychology 58:644. https://doi.org/10.1037/0022-3514.58.4.644

2. Collins NL (1996) Working models of attachment: Implications for explanation, emotion, and behavior. Journal of personality and social psychology 71:810-832. https://doi.org/10.1037/0022-3514.71.4.810

3. Ravitz P, Maunder R, Hunter J, Sthankiya B, Lancee W (2010) Adult attachment measures: A 25-year review. Journal of psychosomatic research 69:419-432. https://doi.org/10.1016/j.jpsychores.2009.08.006

4. Gratz KL, Roemer L (2004) Multidimensional assessment of emotion regulation and dysregulation: Development, factor structure, and initial validation of the difficulties in emotion regulation scale. Journal of psychopathology and behavioral assessment 26:41-54. https://doi.org/10.1023/B:JOBA.0000007455.08539.94

5. Gratz KL, Paulson A, Jakupcak M, Tull MT (2009) Exploring the relationship between childhood maltreatment and intimate partner abuse: Gender differences in the mediating role of emotion dysregulation. Violence and Victims 24:68­-82. https://doi.org/10.1891/0886-6708.24.1.68

6. Kessler RC, Andrews G, Colpe LJ, Hiripi E, Mroczek DK, Normand S-L, et al (2002) Short screening scales to monitor population prevalences and trends in non-specific psychological distress. Psychological medicine 32:959-976. https://doi.org/10.1017/S0033291702006074

7. Smallbone SW, Dadds MR (1998) Childhood attachment and adult attachment in incarcerated adult male sex offenders. Journal of interpersonal violence 13:555-573. https://doi.org/10.1177/088626098013005001

8. Dalton III WT, Frick-Horbury D, Kitzmann KM (2006) Young adults' retrospective reports of parenting by mothers and fathers: Associations with current relationship quality. The Journal of general psychology 133:5-18. https://doi.org/10.3200/GENP.133.1.5-18

9. Robinson CC, Mandleco B, Olsen SF, Hart CH (2001) The parenting styles and dimensions questionnaire (PSDQ). Handbook of family measurement techniques 3:319-321.

10. Baumrind D (1971) Current patterns of parental authority. Developmental psychology 4(1):1-103. https://doi.org/10.1037/h0030372

11. Tagliabue S, Olivari MG, Bacchini D, Affuso G, Confalonieri E (2014) Measuring adolescents' perceptions of parenting style during childhood: psychometric properties of the parenting styles and dimensions questionnaire. Psicologia: Teoria e Pesquisa 30:251-258. https://doi.org/10.1590/S0102-37722014000300002

12. Hawes DJ, Lechowicz M, Roach A, Fisher C, Doyle FL, Noble S, et al (2021) Capturing the developmental timing of adverse childhood experiences: The Adverse Life Experiences Scale. American Psychologist 76:253-267. https://doi.org/10.1037/amp0000760
